# Shortening shift’s length—Should we ask the residents if this is what they want?

**DOI:** 10.1371/journal.pone.0272548

**Published:** 2022-08-02

**Authors:** Yehuda Hershkovitz, Adi Rasco, Orna Tal

**Affiliations:** 1 Faculty of Medicine, Department of Surgery, Shamir Medical Center, Zeriffin, Tel Aviv University, Tel Aviv, Israel; 2 Department of Management, Medical Management program, Bar Ilan University, Ramat Gan, Israel; 3 Faculty of Medicine, Oncology Institute Shamir Medical Center, Zeriffin, Tel Aviv University, Tel Aviv, Israel; 4 Faculty of Medicine, Shamir Medical Center, Zeriffin, Tel Aviv University, Tel Aviv, Israel; VA Boston Healthcare System, UNITED STATES

## Abstract

**Introduction:**

Work overload in hospitals enforced reducing shifts length of physicians in many countries over the last decade. In Israel, the current shift standard is of 26 hours, however, there is a residents’ struggle alongside a governmental intent to short the shifts to 16 hour. We aim to evaluate residents and interns support and preferences regarding shortening shifts and their ramifications to quality of life and residency programs.

**Methods:**

A structured questionnaire was distributed to all resident and interns in a single center. We evaluated their current quality of residency and life, their support in the shorter shifts model, offering alternative program components such as reduced pay, longer residency or replacement in order to allow rest. We compared those who support the new model to those who objected to identify common characteristics to draw a resident profile for acceptance of change.

**Results:**

Overall, 151 physicians answer the questionnaire. 70.2% support the shorter shifts model. Residents above 35 years old and those reaching completion of residency, significantly less support the shortening shifts model. No other demographic nor professional parameters were different between the supporters and non-supporters. Option of reduced pay or longer residency dramatically reduced the support rate to less than 30% and 20%, respectively. Replacement by other physician (resident or senior physician) in order to allow rest was supported by only 40%.

**Conclusion:**

Residents’ standpoints regarding a desirable change are crucial to plan a successful implementation. A national survey is required before a new model is introduced, to achieve an optimal transparent efficient process.

## Introduction

Physicians worldwide are trained in a stressful, overloaded hospital environment prone to error. Medical managers struggle to balance the need for cumulative experience required, shortage of professional manpower to provide sufficient care and enabling residents’ wellbeing. The correlation of medical errors and physicians’ burnout is described in several health systems [1,2]. Attempts to cope with the complexity of reducing errors alongside achieving better quality of life, yet preserving adequate training, yielded only few practical solutions [3].

The effect of shortening shift’s length had been researched in the past two decades, under the rationalization that shortening shift’s length will improve patient’s safety. Additionally, the growing awareness of the "Generation Y" for self-well-being is speeding up the process. Although the need for a change is justified, concerns were raised that the decreased working hours might affect the resident’s education due to the reduction in the physician’s exposure. Bolster et al. in a systematic review article showed that the reduced shift length improved patient care modestly, improved resident wellness but had negative affect on resident’s education [4].

In Israel, shifts last 24–26 hours after which there is 20–22 hours break. Averagely, physician work between 58–76 hours weekly (depend on the number of shifts per month). The workload, which is unparalleled in the job market, is leading to impaired quality of life and can lead to burnout, mental difficulties up to anxiety disorder [5]. While the average hours per week is above the norms in the European Union it is below the average in USA, Canada and South Korea [6].

The detailed rationale for our study was an ongoing deliberation in the Israeli health system considering the length of shifts. Many actors, including the minister of health, the general director, hospital managers and the leading national medical management, declaring it is clear that the shift lengths are too long, yet, no one applied residents to reveal their viewpoint concerning the preferred alternative solution. Our hospital, the 4ht largest hospital in the public governmental hospital net was chosen as a pilot site to assess the benefit of the new solution. Therefore is was extremely interesting to reveal the standpoints of the "main actors" that will play role in the final policy decision. More than 200 resident works in Shamir medical center in Israel in different departments. While some support the straggle, other are more concerned about the impact of this change on their medical education. Shamir MC is a 900 bed general public hospital that provides ~10% of governmental health services for hospitalized patients. We assume similar conditions exist in other general public hospitals.

In this study we aim to evaluate the beliefs of our residents regarding the suggested model of shortening shifts to 16 hours (considering that the physician on duty does not work the morning of his shift). The study was conducted prior to ministerial decision on the subject.

## Methods

This study was approved by the institution’s research ethics committee (protocol 276–21). Study population included residents and interns from a single medical center, who volunteer to participate. The study was performed by a structured questionnaire that was distributed via digital platform (what’s-up). The questionnaire was answered anonymously. Study period was 1 month (November 2021).

The questionnaire included 20 questions and none was mandatory. The questions included medical expertise level, residency level, work load parameters, satisfaction, present wellness evaluation and demographical parameters. We also included Generalized Anxiety disorder– 2 (GAD-2) questions [7]. We based our questions on a suggested plan discussed by the Ministry of Health.

The primary endpoint was the level of support in the reduced shift’s hours. In this suggested new model, the working physician does not work in the morning of his shift, practically, he is working 8 hours less. The secondary end points were level of support in optional shifts models (reduced payment due to reduced work hours, longer residency to compensate for reduced exposure, replacement by senior or resident during the shift in order to allow rest). In questions regarding the support of the new models the scale was from 1–10. We considered score 7 and above as supportive in the offered model.

The questionnaire was evaluated and validated by a steering committee of a surgical representative, internal medicine representative and management level representative. All representatives established the clarity before distribution of the questionnaire.

In the analysis, physicians in internal medicine departments, neurology, emergency medicine, pediatrics and imaging were included in the medical ward. Physicians in general surgery, orthopedics, OBGYN, ENT, urology, ophthalmology, Anastasia and pediatric surgery were included in the surgical ward.

We divided residency period into two: "senior residents" that are in their last 2 years of residency and "junior residents" that have more than 2 years to finalization.

### Statistical analysis

The association between categorical variables was assessed using either the Chi-square or the Fisher’s exact tests. All statistical tests applied were two-tailed, and a p-value of ≤0.05% was considered as significant.

## Results

Overall 151 physicians answer the questionnaire. There were 122 resident (which account for 57% for our medical center residents) and 29 interns (which account for 32% for our medical center interns). There were 66 (43.7%) residents from surgical wards, 56 (37.1%) residents from medical wards. 53.8% were male and 46.2% female. 61.9% were under 35 years old. 57.1% are in relationship with children, 21.8% in relationship without children, and 21.1% singles.

Overall, 106 physicians (70.2%) support the new reduced shift model, 73.2% of medical ward residents, 68.2% of surgical ward residents and 71.4% of interns. The differences were not significant. Residents in the last part of their residency significantly support less in the new model (55% vs. 78.8%, p<0.05). The younger the resident, the more he support the new model. Resident under 35 significantly support more in the new model (p<0.05). Residents who feel that the quality of the residency is not good, tend to support more in the new model (76.8% vs 67.7%, P = 0.26).Current number of shift per month conducted by the participant didn’t affect the support in the new model. Self-evaluation of quality of life, GAD-2 score and insufficient time to research were not significantly different in those who support the new model. Not gender nor marital status affect the support in the new model.

Third of the physician think the new model will improve the quality of the residency and third think it will be damaged. 87.3% believe their quality of life will be improved. 57.3% predict increased in workload during the morning shift (for themselves and their peers) due to shortage of personal.

Compression of professional and demographic characteristics between supporters (7–10) and non-supporters (1–6) of the new shift model is detailed in Table 1.

**Table 1:** Compression of professional and demographic characteristics between supporters and non-supporters of the new shift model.

			Non to partial supportive 1–6	Support to very supportive 7–10	P value
Professional parameters	Profession	Medical ward	15 (26.8%)	41 (73.2%)	0.82
		Surgical ward	21 (31.8%)	45 (68.2%)	
		Interns	8 (28.6%)	20 (71.4%)	
	Residency stage	Pre-residency (interns)	8 (28.6%)	20 (71.4%)	0.026
		Junior residents	17 (21.3%)	63 (78.8%)	
		Senior residents	18 (45.0%)	22 (55.0%)	
	Quality of residency	Good	30 (32.3%)	63 (67.7%)	0.26
		Not good	13 (23.2%)	43 (76.8%)	
	Number of shifts	6 or less	36 (30.0%)	84 (70.0%)	0.82
		More than 6	8 (26.7%)	22 (73.3%)	
	Research ability	Damaging	28 (26.7%)	77 (73.3%)	0.23
		Not damaging	16 (37.2%)	27 (62.8%)	
Demographic parameters	Age groups	<35	21 (48.8%)	70 (68%)	0.039
		35 and above	22 (51.2%)	33 (32%)	
	Gender	Male	26 (33.8%)	51 (66.2%)	0.205
		Female	16 (23.9%)	51 (76.1%)	
	family status	Single	8 (25.8%)	23 (74.2%)	0.403
		Relation without children	7 (21.9%)	25 (78.1%)	
		Relation with children	28 (33.7%)	55 (66.3%)	
	Quality of life	Good	7 (22.6%)	24 (77.4%)	0.38
		Not good	37 (31.4%)	81 (68.6%)	
	GAD-2 anxiety disorder	Less than 3 (no anxiety)	23 (35.4%)	42 (64.6%)	0.21
		3 or more (anxiety)	21 (25.0%)	63 (75.0%)	

**Fig 1:**
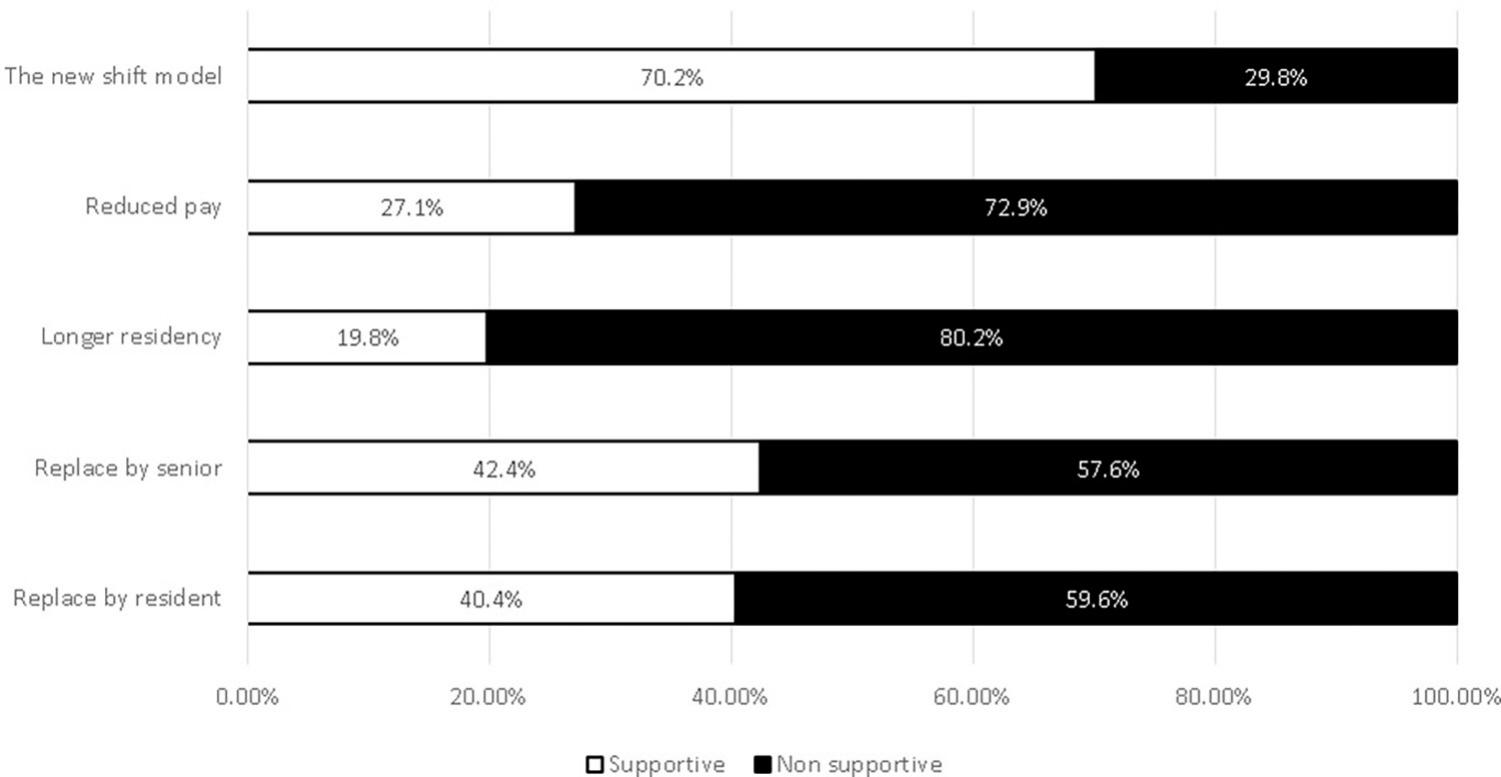


The support in the new model changed dramatically when pay reduction was considered (41 physicians– 27.1%) or with elongation of the residency (30 physicians– 19.8%). Other options of replacement in order to rest by senior or by resident were supported by 42.4% and 40.4% respectively (Fig 1).

## Discussion

In Israel, the number of doctors per 1000 population is 3.19, which is lower than the OECD average of 3.5 [8]. Prediction for 2035 yields a continuous reduction of physicians per population [9]. The average length of residency is 5 years, the residents are required to work at least 4–6 night shifts per month. In accordance with specific department needs, the number of shifts might go up to 8–10 per month. Shifts last 24–26 hours after which there is 20–22 hours break. On average, a resident works between 58–76 hours per week (depending on the number of shifts per month).

During the past decade many countries had implemented regulation to working hours of residents. The US Accreditation Council for Graduate Medical Education (ACGME) has set a cap of 80 h/week, and also a mandate on the amount of sleep/personal time for residents (10 h between shifts), as well as the maximum length of shifts (16 h with 30 h maximums), and the number of overnight calls (no more than once every 3 days). The European Union working time directive has also set 48 h as the maximum limit. These restrictions, however, generated criticisms with regard to the resulting lower volume of experience and slower acquirement of higher skills [10]. New regulations for working hours of medical doctors implemented in Austria during 2018, based on the European directive 2003/88/EG, limiting on-duty working hours to 48 h per week. In a survey conducted by Bergmeister over 50% of doctors and medical students are still undecided whether reduced work hours may also positively influence medical education [11]. In Sweden, it was found that even a 16-hour night-call schedule required two nights sleep recovery [12]. In Taiwan the average total work hours per week of an attending physician is around 69.1 h, but duty shifts differ among hospital accreditation levels, geographic locations, emergency care responsibilities, and medical specialties. They recommend adjusting physician work hours to a reasonable level [13].

Jagsi and Suender [14] evaluate the experience and attitude of physicians from UK and US after the regulation of hours took effect (the regulation match the status in Israel without the new model). Their main conclusion was that reducing working hours without investment of resources and redesigning the training system, will not result in the intended improvement. Later study also report similar results [15]. In South Korea, the Medical Resident Act took place in 2015 and for the first time restricted the working hours of residents to 80 hours per week, also defining residency programs and directors. In a study that evaluated the effect of the act on residents, they report improvement in learning environment and satisfaction from the residency but show increase in workload and less lunch breaks [16].

Our study is unique, since it evaluates the opinion of residents before the desired regulation took place.

Most of the residents in our study are part of generation Y, the Millennials. This generation put work as secondary priority after life quality and pursuit of happiness. The concept of "Life work balance above all else" can best describe the setoff mind of the Millennials. They believe that happy life lead to better productivity. Lafraia et al, found that less than 50% of the surgical resident are happy at work regardless to their enjoyment in the operation theater [17]. Moreover, residents at the present time are sensitive to fairness in the distribution of shifts, and this may influence their productivity and satisfaction [18].

Understanding the priorities of nowadays residents is a main concern of residency program directors who required to adapt changes in order to attract residents [19–21]. Our study shows that the older or more senior the trainee is, the less likely he/her supports the change. Possible explanation is the millennial approach toward changes and work balance. Other explanation might be related to the responsibilities differences between senior and junior residents. Senior resident enjoys privileges that they fear to lose because of the changed model.

It appears interns are no different in their professional perspective regarding the effect of long working hours. Landrigan at el. [22] reported higher rates of serious errors are performed by internes while working 24 hour shifts every third night. In another study, after intervention (reducing work hours) Lockley et al. [23] reported increased overall sleep hours and decreased attentional failures during night work hours. Barger et al. reported increased risk of motor vehicle accidents in internes performing extended shifts [24].

In Israel, interns work conditions are very strict. There are 4 shifts per month (24–26 hours) which are mandatory with optional 2 more shifts voluntary. In our study 29 of the participants were interns, which account for 30% of overall interns in our institute. 62.1% of them worked only the 4 mandatory shifts. 70% of the interns support the new shift model however surprisingly enough, 40% of those who support the new model, choose to do voluntary shifts. The options of reduced payment per shift or elongate residency caused a change in the declared preferences, as 80% do not support the change. Since the work of interns is supervised closely and they are not allowed to sign medical orders, their work load and responsibilities are less than their resident peers. Nevertheless, their aspirations for better working condition are the same.

Analyzing our findings, (Fig 1) revealed significant differences between the high supports of shifts shortening when it is an isolated conceptual idea without consequences, and the much lower support when there is a need for a sacrifice. Shortening of shifts that have been supported by 70% of the respondents, dropped to 30% once being asked if the shorter shifts will carry a cost, such as lower salary, and even to 20% if a longer residency has been suggested. Other options such as partial replacement by attending or another resident in order to allow rest also didn’t get a lot of support (Fig 1). The reason might be that the residents don’t perceive this option as improvement to their quality of life since it will require their presence in the hospital after hours (as residents currently, or as seniors in the coming years). Another reason is that replacement for only few hours does not reduce the accountability and responsibility of the resident on call and as such the "rest" is not really possible.

In our cohort, only 20 physicians (13.2%) support the new model even in the cost of reduced pay or longer residency. This group have no common characteristics, except for trend to anxiety (75% vs.53.8%, P = 0.09). There were no differences in other professional or demographical parameters.

As this topic is recently deliberated nationwide, our colleagues also investigated suggested models to decrease the duty hour length and night shift frequency. They concluded short 16 h shifts would have a positive effect on the balance between personal life and work, not impairing training during residency. Similar to our findings, in their research 74% of the residents were not willing to lower their income if the decision were made to change models, and 56% were not willing to increase the number of shifts [25].

In Israel most residents are represented by Israel Medical Association (I.M.A) (which also represents all senior physicians) and less than 10% of residents are represented by a relatively new Medical Association -MIRSHAM. In our study, 40% of responders are represented by IMA, 21% by MIRSHAM and the rest did not declare being represented by any professional union. Rates of supporters of the new model were 75.4% from IMA and 65.6% from MIRSHAM. Byju and Mayo [26] describe the ethical and empirical criteria that justify collective action as a union. They discuss "the free rider", the one who benefit from the fruits of the union work without contributing to the union, meaning those that do not want or support the change, but it is forced on them. Shortening the shifts even without changing the payment nor changing the residency length, has significant financial ramifications to the hospital. It demands increase in manpower that might not be available especially in the smaller hospitals in the periphery. Those significant changes that are required will affect the whole medical profession and there could not be differentiation between the supporter and non-supporter. This is why, the decision should reflect the wishes of most residents. These forces in the healthcare area may represent political incentives that only partially correlate with residents’ preferences as individuals.

The public opinion plays a significant rule in ministerial decision processes. The recent Covid-19 pandemic around the world and in Israel increased the awareness to the physician’s work load. The influence of public opinion is not well discussed in the literature. Blum et al. conducted a survey to evaluate public opinion on resident working hours, showed overwhelmingly support in shortening shifts from 30 hours and strong support in restricting it to 16 hours [27]. While this question is beyond the scope of our study, as long as the struggle is perceived as an effort to improve patient safety and care, it will have extensive public support. However, our findings advocate that the primary motive for the desired change is the physician’s wellbeing and as such, one cannot predict public opinion and support in the struggle.

Reviewing attempts in other countries to solve this issue, many look forward to implementing guidelines or regulations to limit physicians’ working hours. We rise the importance of engaging residents, the actual players, to assess the potential success of alternative solutions. This is an innovative study that presents possible models of shorting shifts, so that the residents have the opportunity to be involved in molding their carriers and express their feelings and preferences in a transparent manner.

Our study has several limitations. The study account for only single center experience, nevertheless, similar findings were published by colleagues from other hospital in Israel. Although the recruitment for the study was relatively high (60% of the residents in our medical center), the sample size is too small to identify differences between subspecialties.

## Conclusion

Since there is high variability in residents’ view on shortening shift’s length we suggest that the residents should be part of the decision process. They should be aware of the ramifications. A national survey should be conducted in order to best represent their opinions.

## Supporting information

S1 Data (XLSX)

S1 Questionnaire (DOCX)
