# Supplementary material for: Shortening shift’s length—Should we ask the residents if this is what they want?
Source: PLoS One. 2022 Aug 2;17(8):e0272548. doi: 10.1371/journal.pone.0272548 (PMC9345332; doi:10.1371/journal.pone.0272548)
Supplement: S1 Questionnaire — (DOCX) [file pone.0272548.s002.docx]

Questionnaire:

What is your opinion on shortening shift's length?

Dear Doctor, this questionnaire aim to evaluate your standpoint regarding the planed model for shortening shift's length.

This questionnaire is anonymous.

Time required for filling the form is 2-4 minutes.

Please continue only if you approve your participation in the study.

1. Age (groups)
2. Gender
3. Marital status (single, in relation, with children).
4. Are you registered in physician union? (IMA, MIRSAM, NONE)
5. Which residency?
6. Stage of residency (first 2 years, middle, last 2 years)
7. Number of shifts per month (number).
8. What is the professional level of your residency, in your opinion (1-10)
9. How do you grade your quality of life (bad 1- excellent 10)
10. To what level your residency hinder your ability to do research? (1-10)
11. During the last 2 weeks, how often did you felt stressed, worried or "on edge"? (not at all, several days, more than half days, almost every day)
12. During the last 2 weeks, how often did you felt that you can't stop worry? (not at all, several days, more than half days, almost every day)
13. In the new shift model it is suggested that the resident will arrive strait to the shift without working the morning hour (reducing shift's length from 24-26 hours to 16-18 hour). How much do you support this change? (1-10)
14. To what level will this change will affect the professional level of the residency? (significant drop, mildly drop, no change, mildly better, significantly better)
15. To what level will this change will affect your quality of life? (significantly worse, mildly worse, no change, mildly better, significantly better)
16. To what level will this change will affect the morning workload? (workload will decrease, no change, increase)
17. If as part of the change, the payment will be decreased (to fit the actual work hours), how much do you support this change? (1-10)
18. If as part of the change, the residency period will be increase, how much do you support this change? (1-10)
19. If alternatively, instead of changing shift's length, a senior physician will be added to the shift's staff enabling 4 hours of rest to each of the physicians on duty. How much do you support this change? (1-10)
20. If alternatively, instead of changing shift's length, a resident will be added to the shift's staff enabling 4 hours of rest to each of the physicians on duty. How much do you support this change? (1-10)

Thank you for answering this questionnaire.

The research team
